# The Leukemic *Isocitrate Dehydrogenase (IDH) 1/2* Mutations Impair Myeloid and Erythroid Cell Differentiation of Primary Human Hematopoietic Stem and Progenitor Cells (HSPCs)

**DOI:** 10.3390/cancers16152675

**Published:** 2024-07-27

**Authors:** Sara Pierangeli, Serena Donnini, Valerio Ciaurro, Francesca Milano, Valeria Cardinali, Sofia Sciabolacci, Gaetano Cimino, Ilaria Gionfriddo, Roberta Ranieri, Sabrina Cipriani, Eleonora Padiglioni, Roberta Iacucci Ostini, Tiziana Zei, Antonio Pierini, Maria Paola Martelli

**Affiliations:** 1 Hematology and Clinical Immunology Section, Department of Medicine and Surgery, Center for Hemato-Oncological Research (CREO), University of Perugia, 06123 Perugia, Italy; sara.pierangeli@unipg.it (S.P.); serena.donnini@dottorandi.unipg.it (S.D.); francesca.milano@unipg.it (F.M.); valeria.cardinali@unipg.it (V.C.); gaetano.cimino@specializzandi.unipg.it (G.C.); ilaria.gionfriddo@unipg.it (I.G.); roberta.ranieri@specializzandi.unipg.it (R.R.); sabrina.cipriani@unipg.it (S.C.); antonio.pierini@unipg.it (A.P.); 2 MD Anderson Cancer Center, University of Texas, TX 78712, USA; v.ciaurro@mdanderson.org; 3 Hematology Department, ‘Santa Maria della Misericordia’ Perugia Hospital, 06129 Perugia, Italy; sofia.sciabolacci@ospedale.perugia.it (S.S.); roberta.iacucci@ospedale.perugia.it (R.I.O.); tiziana.zei@ospedale.perugia.it (T.Z.)

**Keywords:** acute myeloid leukemia, *IDH1/2* mutation, human HSPC modeling

## Abstract

**Simple Summary:**

Acute myeloid leukemia (AML) arises from a stepwise acquisition of multiple genetic alterations in the hematopoietic stem and progenitor cell and is characterized by an accumulation of immature leukemic cells, called blasts, in the bone marrow and tissues. How the single gene alterations mediate the underlying processes is unclear in the majority of cases. *IDH1/2* mutations are among the most frequent mutations in AML, accounting for about 15–20%, and are targets of specific small molecule inhibitors. Experimental evidence suggests that they occur early in leukemogenesis. Mostly, murine models of *IDH1/2* mutations have been studied. Our study is the first to highlight that—in the human system—*IDH1/2* mutants drive a complete block of hematopoietic cell differentiation in vitro, which is completely unleashed by the specific inhibitor, providing a model for early leukemogenesis studies in this setting.

**Abstract:**

How hematopoietic stem and progenitor cell (HSPC) fate decisions are affected by genetic alterations acquired during AML leukemogenesis is poorly understood and mainly explored in animal models. Here, we study *isocitrate dehydrogenase* (*IDH*) gene mutations in the human model of HSPC and discuss the available literature on this topic. *IDH1/2* mutations occur in ~20% of AML cases, are recognized among the mutations earliest acquired during leukemogenesis, and are targets of specific inhibitors (ivosidenib and enasidenib, respectively). In order to investigate the direct effects of these mutations on HSPCs, we expressed *IDH1*-R132H or *IDH2*-R140Q mutants into human CD34+ healthy donor cells via lentiviral transduction and analyzed the colony-forming unit (CFU) ability. CFU ability was dramatically compromised with a complete trilineage block of differentiation. Strikingly, the block was reversed by specific inhibitors, confirming that it was a specific effect induced by the mutants. In line with this observation, the CD34+ leukemic precursors isolated from a patient with *IDH2*-mutated AML at baseline and during enasidenib treatment showed progressive and marked improvements in their fitness over time, in terms of CFU ability and propensity to differentiate. They attained clonal trilinear reconstitution of hematopoiesis and complete hematological remission.

## 1. Introduction

Acute myeloid leukemia (AML) arises from genetic abnormalities in hematopoietic stem or progenitor cells (HSPCs), and it is responsible for uncontrolled growth and accumulation of neoplastic blasts in bone marrow (BM). AML is an aggressive, clinically and biologically heterogeneous disease, in which a large number of recurrently mutated genes have been identified [1]. A stepwise acquisition of more than one alteration is required for leukemia development, although it is not always clear how single mutations contribute to leukemogenesis [2,3].

Mutations in *Isocitrate Dehydrogenase* (*IDH*) 1 and 2 were originally identified in glioma [4,5], AML, myeloproliferative neoplasm, myelodysplastic syndrome patients [6,7,8], and in other solid tumors [9,10]. In AML, approximately 20% of patients carry these mutations, especially in the normal karyotype setting [1,11]. *IDH1* mutations occur thereabout in 7−10% of patients, while 10−15% are *IDH2*-mutated, and their frequency increases with age [6,12,13,14].

Isocitrate dehydrogenases are homodimeric enzymes that play an important role in cellular metabolism and the regulation of the response to hypoxia [15]. Physiologically, the isoform IDH1, localized in the cytoplasm and peroxisomes, is involved in lipid and carbohydrate metabolism, providing NADPH to cells and protecting them from reactive oxygen species. IDH2, on the other hand, is localized in the mitochondrial matrix and participates in the tricarboxylic acid cycle to protect cells from oxidative damage. Both isoforms catalyze the oxidative decarboxylation of isocitrate, generating α-ketoglutarate (αKG) and carbon dioxide (CO_2_), and producing reduced NADPH from NADP+, a process that is essential for preserving cellular homeostasis [16].

In AML, the *IDH* mutations are heterozygous and missense. Aminoacidic substitution in IDH1 most often involves arginine 132 with cysteine or histidine (R132C or R132H). In IDH2, arginine is replaced by glutamine at residue 140 (R140Q) and by lysine at residue 172 (R172K) [16,17,18,19]. These mutations result in gain-of-function neomorphic activity that allows the IDH enzymes to catalyze the reduction of αKG to the oncometabolite (R)-2-hydroxyglutarate (2-HG) [20,21,22]. Abnormal accumulation of 2-HG has been shown to inhibit αKG-dependent dioxygenases as the ten–eleven translocation methylcytosine dioxygenase 2 (TET2), dysregulating the epigenetic machinery of hematopoietic progenitors; this may explain why *IDH1* and *IDH2* mutations are mutually exclusive with those affecting *TET2* [20,23,24]. On the other hand, *IDH1/2* mutations are frequently associated with *nucleophosmin* (*NPM1*) gene mutations and internal tandem duplication in *FMS-like tyrosine kinase 3* (*FLT3*-ITD) [12,25].

The bona fide correlation between mutations and leukemogenic mechanisms has prompted intense drug discovery, targeting the mutant IDH. In particular, two first-in-class small molecule inhibitors have been approved by the FDA: enasidenib (AG-221) and ivosidenib (AG-120) [26,27]. Enasidenib is a selective allosteric inhibitor of the IDH2 mutation: its function involves the binding and stabilization of the open conformation of the IDH-mutated enzyme, and, primarily, the inhibition of the conversion of αKG to 2-HG2 [19,28]. Ivosidenib is a reversible, allosteric competitive inhibitor of the mutated IDH1. Ivosidenib competes for binding with the magnesium ion, an essential cofactor for the IDH1 mutant, thereby preventing the formation of a catalytically active site [19,28].

*IDH1/2* mutations and the other genetic lesions in genes encoding for epigenetic modifiers (*DNMT3A*, *ASXL1,* and *TET2*) are early events in the development of AML, like those observed in deep sequencing and single-cell studies determining clonal evolution in myeloid malignancies [11,29]. These mutations are typically found in the founding clone of AML and are rarely found alone [11], suggesting that they are not sufficient to drive leukemia. Effectively, numerous studies in mouse models have demonstrated that *IDH* mutations alone cannot cause overt leukemia in vivo [30,31] but cooperate with additional genetic lesions to initiate cancer [32,33,34,35,36,37,38]. The direct effects of single *IDH* AML-associated mutations on human HSPCs are poorly studied, so it remains unclear how they affect cell fate decisions, promote leukemogenesis, and contribute to the maintenance and progression of the malignant phenotype. Although some mouse model studies have elucidated how *IDH* mutations affect mouse HSPCs [30,31,32,33,34], specific studies in humans are needed to address the species-specific differences in basic biology and hematology.

Here, in order to investigate the direct effects of these mutations on the HSPC, we express *IDH1*-R132H or *IDH2*-R140Q mutants into human CD34+ cells from healthy donors via lentiviral transduction; we analyze changes in their hematopoietic fitness using the colony-forming unit (CFU) assay, which allows measuring the proliferation and differentiation abilities of individual hematopoietic stem cells and evaluates the effects of specific inhibitors, ivosidenib and enasidenib, respectively. Moreover, we studied the fitness dynamics of CD34+ cells isolated directly from a patient with *IDH2*-mutated AML undergoing treatment with the IDH2-inhibitor enasidenib.

## 2. Materials and Methods

### 2.1. Human CD34+ Hematopoietic Stem and Progenitor Cells from Healthy Donors

Human CD34+ HSPCs (hCD34+) derived from mobilized peripheral blood of n = 13 healthy donors (HD) (Supplementary Table S1) were obtained upon written informed consent. This study was conducted according to the Declaration of Helsinki and approved by the local ethics committee (protocol code: 2018-07). After mobilizing with rHuG-CSF (Lenograstim), peripheral blood cells were selected for CD34 marker expression with CliniMACS^®^ technology (Miltenyi Biotec Inc., Auburn, CA, USA), according to standard procedures followed at our institute [39]. Leftover cells after infusion to the patient were recovered for the experiments. Cells were stained with APC/Cyanine7 anti-human CD34 antibody and FITC anti-human CD38 antibody (BioLegend^®^, San Diego, CA, USA) and analyzed by Fluorescence Activated Cell Sorting (FACS) using BD FACSCanto II^TM^ (Becton, Dickinson, Franklin Lakes, NJ, USA) to assess immunophenotype. For each donor, up to 10 × 10^6^ CD34+ were cultured in StemSpan™ SFEM II serum-free medium (STEMCELL^TM^ Technologies, Vancouver, BC, Canada) supplemented with penicillin/streptomycin (1:100), recombinant human FMS-related tyrosine kinase 3 ligand/FLT-3 Ligand (100 ng/mL), stem cell factor/SCF (100 ng/mL) and recombinant human thrombopoietin/TPO (50 ng/mL) (R&D SYSTEMS, Minneapolis, MN, USA) for 48 h before cell transduction (hereafter, named “HSPC-retention medium”).

### 2.2. Sequences and Expression Vector

Wild-type coding sequences used for lentiviral transduction are as follows:

- Homo sapiens isocitrate dehydrogenase (NADP(+)) 1, cytosolic (IDH1), transcript variant 1, mRNA (NCBI reference sequence: NM_005896.3).
- Homo sapiens isocitrate dehydrogenase (NADP(+)) 2, mitochondrial (IDH2), transcript variant 1, mRNA (NCBI reference sequence: NM_002168.3).
- Mutated sequences were virtually designed according to the most common nucleotide change in cytogenetically normal–AML [17]:
- IDH1-R132H (nucleotide change c.395G>A).
- IDH2-R140Q (nucleotide change c.419G>A).

All sequences were synthesized and provided by GenScript^®^ (Piscataway, NJ, USA) into the pLVX-EF1α-IRES-ZsGreen1 (Takara Bio, Shiga, Japan) vector, in which a bicistronic RNA is transcribed by the same promoter and the internal ribosome entry (IRES) site allows for internal/cap-independent translation initiation, obtaining the coordinated expression of genes of interest (GOIs) and the reporter gene (ZsGreen1).

### 2.3. Lentivirus Production

The HEK 293T packaging cell line was cultured in DMEM supplemented with 10% FBS, 1% penicillin/streptomycin, and 1% glutamine at 37 °C in a 5% CO_2_ atmosphere. For lentiviral particle production, HEK 293T cells were co-transfected by Lipofectamine^TM^3000 (Invitrogen™-Fisher Scientific Inc., Waltham, MA, USA), according to the manufacturer’s instructions, using a second-generation lentiviral system. This system consists of the expression vector pLVX-EF1α-IRES-ZsGreen1, which contains the GOIs *IDH1* wild-type/R132H and *IDH2* wild-type/R140Q, in combination with packaging plasmids, psPAX2, a gift from Didier Trono (Addgene plasmid no. 12260; http://n2t.net/addgene:12260 (accessed on 24 July 2024); RRID: Addgene_12260), and BaEV-TR345, kindly granted by Els Verhoeyen (Université de Lyon, Lyon, France) [40]. The viral supernatants were collected 48 h after transfection and concentrated 100× by centrifugation at 3000× *g* for 16 h at +4 °C. The viral titer was estimated by transducing HEK 293T cells with serially diluted vectors and quantifying the proportion of ZsGreen1+ cells by FACS.

### 2.4. hCD34+ Lentiviral Infection

Lentiviral infections of CD34+ primary cells were conducted according to the RetroNectin^®^ (Takara Bio Inc., Kusatsu, Japan) protocol guidelines, which were optimized for our needs. For each well of a 6-well plate, 2 mL of growth HSPC-retention medium was added along with lentiviral 100× concentrate supernatant of *IDH1* wild-type/R132H, or *IDH2* wild-type/R140Q, or an empty vector at a multiplicity of infection = 30. The experimental conditions are illustrated in Supplementary Table S1. The plate was centrifuged at 2000× *g* for 2 h at 32 °C for virus pre-loading. The target cells CD34+ were counted and resuspended in HSPC-retention medium at 2 × 10^6^/mL and 1 mL per well was added. The plate was then centrifuged at 1800 RPM for 45 min at 32 °C and then incubated at 37 °C in a 5% CO_2_ atmosphere for 72 h. 

### 2.5. hCD34+ Cell-Staining and Sorting Strategies

After incubation, cells were harvested, washed with PBS, and stained for 20 min in the dark at RT with APC/Cyanine7 anti-human CD34 antibody. Cells were re-suspended in PBS, HSA (0.5%), and EDTA (2 mm) before sorting. An unstained control was made to set up forward and side scatter parameters on the cell sorter and to set positive gates. The cells were sorted by FACS using a BD FACSAria™ III cell sorter (Becton Dickinson, Franklin Lakes, NJ, USA). Transduced bright ZsGreen1+/CD34+ double-positive cells expressing either the empty vector as the control, *IDH1/2* WT, or *IDH1*-R132H/*IDH2*-R140Q were used for CFU assays in methylcellulose medium. In our 13 human CD34+ cell samples transduced with GOIs, we achieved ZsGreen1 reporter gene cell positivity. The sorting strategy is depicted in Supplementary Figure S1.

### 2.6. hCD34+ Cells, Colony-Forming Unit Assays

**Figure 1:**
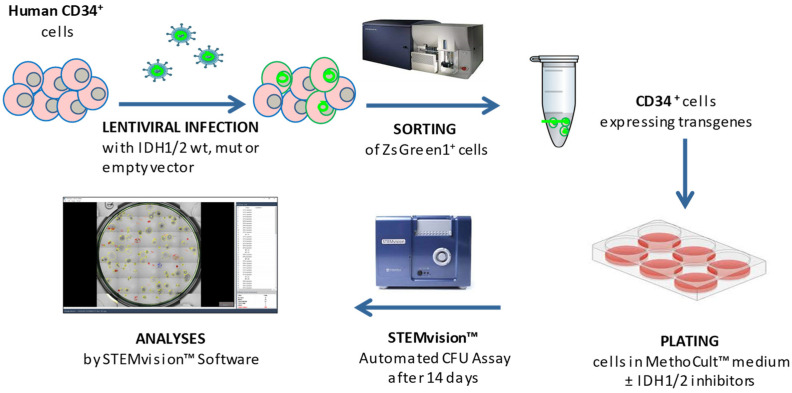
Experimental workflow.

CFU assays were performed by culturing hematopoietic cells in MethoCult™ H4435 Enriched (STEMCELL^TM^ Technologies, Vancouver, BC, Canada) in accordance with the manufacturer’s instructions. After sorting, cells were re-suspended in 300 μL of SFEM II and added to MethoCult™ H4435 enriched in the presence or absence of the specific inhibitor ivosidenib (AG-120, 5 μM) or enasidenib (AG-221, 5 μM) (Selleckchem, Houston, TX, USA) (Supplementary Table S1). Cells were then plated in meniscus-free SmartDish™ cultureware at a density of 500 cells/well in different wells and cultured for 14 days at 37 °C and 5% CO_2_; 4 wells for each sample condition have been foreseen for subsequent statistical analyses. Colonies were counted and characterized by subtype after 14 days of culture using the STEMvision™ instrument and its analysis software STEMvision Colony Marker (https://www.stemcell.com/products/brands/stemvision-automated-imaging-colony-assay.html, accessed on 24 July 2024; STEMCELL™ Tech, Vancouver, BC, Canada). The subtypes included colony-forming unit–Erythroid (CFU-E), burst-forming unit–Erythroid (BFU-E), colony-forming unit–granulocyte, macrophage, granulocyte/macrophage (CFU-G/M/GM), and colony-forming unit–granulocyte, erythrocyte, macrophage, and megakaryocyte (CFU-GEMM). The experimental workflow is illustrated in Figure 1.

### 2.7. CD34+ Cells from a IDH2-Mutated AML Patient

Primary cells from a patient with *IDH2*-mutated R140Q AML were obtained, upon written informed consent, at the Hematology Institute of Perugia (Italy). The study was conducted according to the Declaration of Helsinki and following a protocol approved by the local Ethics Committee. Samples were obtained from BM aspirates performed at diagnosis and under therapy with enasidenib (AG-221) at 100 mg/die (for 28 days = 1 cycle), administered as third-line chemotherapy, on day 0 (before starting the treatment), and after the I, II, and IV therapy cycles. Mononuclear cells were separated by Lymphoprep™ (Serumwerk Bernburg AG for Alere Technologies AS, Oslo, Norway) according to the manufacturer’s protocol. APC/Cyanine7 anti-human CD34 antibody staining was performed and CD34+ cells were isolated by the cell sorter. Sorted CD34+ AML cells were plated in MethoCult™-enriched medium for CFU assays, and incubated for 14 days at 37 °C, in a 5% CO_2_ atmosphere, with images captured by STEMvision™ as described above. 

### 2.8. Next-Generation Targeted-DNA Sequencing (NGS)

Determination of the *IDH2*-mutant allele frequency was carried out by extracting DNA from the primary *IDH2*-R140Q AML BM sample (bulk, myeloid, erythroid, and CD34+ cells) at the fourth cycle of enasidenib therapy. The DNA sample collected for diagnostic procedures was used for DNA sequencing analysis to obtain a detailed overview of DNA mutations in our AML patient. The DNA concentration was assessed using a fluorometric quantitation via the Qubit 3.0 fluorometer (Thermo Fisher Scientific cat. no. Q33216, Waltham, MA, USA), while the DNA integrity numbers (DINs) were evaluated by microfluidic electrophoresis on an Agilent TapeStation using Agilent High Sensitivity D1000 ScreenTape System (Agilent, Santa Clara, CA, USA). The Qiagen Myeloid Neoplasm sequencing panel (Qiagen, Germantown, MD, USA, cat. nr. 333502) was used to prepare libraries using 40 ng of DNA from each sample according to the manufacturer's instructions. The sequencing process was carried out on the MiSeq Illumina platform, generating 150 base-paired end reads. Variant calling was performed using the GeneGlobe online data analysis platform (Qiagen, USA). 

### 2.9. Statistical Analysis

For the transduced hCD34+ CFU assay, technical replicates of 4 wells for each condition have been foreseen and subsequent statistical analysis was performed on the mean of a minimum of 3 wells per sample. A graphical representation of data was performed using GraphPad Prism [v7]. Statistical analyses were performed by the one-way ANOVA test (**** *p* < 0.0001, *** *p* < 0.001, ** *p* < 0.01, * *p* < 0.05).

## 3. Results

### 3.1. Either the IDH1-R132H or IDH2-R140Q Mutation Blocks CFU Ability of Human CD34+ HSPC

To investigate the direct effects of recurring AML *IDH1/2* mutations, hCD34+ cells from HD were transduced by a lentiviral expression vector, containing either *IDH1* wild-type or *IDH1*-R132H, along with a ZsGreen1 reporter gene (n = 7 donors), or containing either *IDH2* wild-type or *IDH2-R140Q*, also with a ZsGreen1 reporter gene (n = 6 donors) (Supplementary Table S1), and sorted as described in the Materials and Methods section.

**Figure 2:**
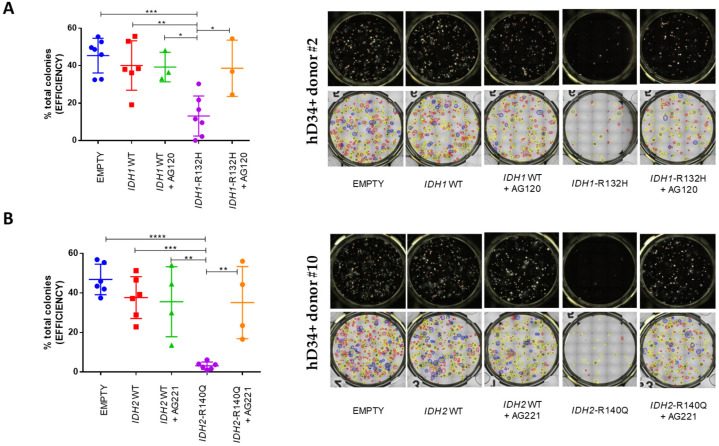
*IDH1*-R132H and *IDH2*-R140Q mutations drive a block of CFU ability in human CD34+ HSPC, which is released by specific inhibitors. The CFU assay of transduced hCD34+ cells with an empty vector, *IDH1* wild-type, or *IDH1*-R132H (n = 7), ±AG-120 inhibitor (n = 3 of 7) (**A**); empty vector, *IDH2* wild-type, or *IDH2*-R140 (n = 6), ±AG-221 inhibitor (n = 4 of 6) (**B**). Graphs on the left represent the percentage of total colonies that rose after 14 days. Panels on the right are representative images of wells (top row) and types of colonies (bottom row) obtained using the STEMvision™. Colony marker application: red circles identify BFU-E-derived colonies; yellow circles identify CFU-G/M/GM-derived colonies; blue circles identify CFU-GEMM-derived colonies; and orange circles identify CFU-E-derived colonies (one-way ANOVA analyses **** *p* < 0.0001, *** *p* < 0.001, ** *p* < 0.01, * *p* < 0.05).

ZsGreen1-positive sorted cells were used for CFU assays, either in the presence or absence of the selective inhibitor AG-120 (n = 3 out of 7 donors for *IDH1* experimental set) or AG-221 (n = 4 out of 6 donors in *IDH2* set) (Figure 2). 

Colonies were assessed at 14 days. In our control groups, cloning efficiency from hCD34+ expressing the empty vector was comparable to hCD34+ bearing *IDH1* wild-type or *IDH2* wild-type and was not significantly influenced by treatment with ivosidenib or enasidenib. Instead, hCD34+ with the *IDH1*-R132H mutation showed an evident block of differentiation compared to hCD34+ transduced with the empty vector (*p* = 0.0002 ***) or wild-type gene (*p* = 0.0025 **) counterparts. This block was even more pronounced with the *IDH2*-R140Q mutation when compared with an empty vector (*p* < 0.0001 ****) or with the wild-type (*p* = 0.0004 ***) exogenous expression. In line with being a specific effect of the *IDH1* or *IDH2* mutations, the block was released, in both cases, by AG-120 (*p* = 0.0257 *) or AG-221 (*p* = 0.0028 **), respectively (Figure 2).

**Figure 3:**
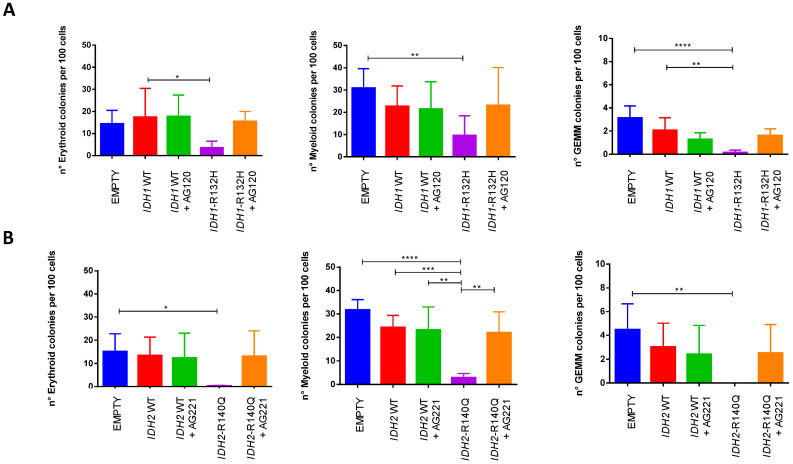
The block of differentiation induced in human CD34+ by either *IDH1* (**A**) or *IDH2* (**B**) mutations and the release by the specific inhibitors affect all lineages: erythroid (left), myeloid (middle), or GEMM (right) precursors (one-way ANOVA analyses **** *p* < 0.0001, *** *p* < 0.001, ** *p* < 0.01, * *p* < 0.05).

Interestingly, no differences in the proportions of erythroid (BFU/CFU-E), myeloid (CFU-G/M/GM), and mixed (CFU-GEMM) colonies were observed in the different conditions for both *IDH-analyzed* mutations, suggesting that the block of differentiation and release induced by the specific inhibitors affect all three lineages (Figure 3).

### 3.2. Enasidenib (AG-221) Treatment Induces a Progressive Improvement of CFU Ability of Primary CD34+ Cells in a Patient with IDH2-Mutated AML

**Figure 4:**
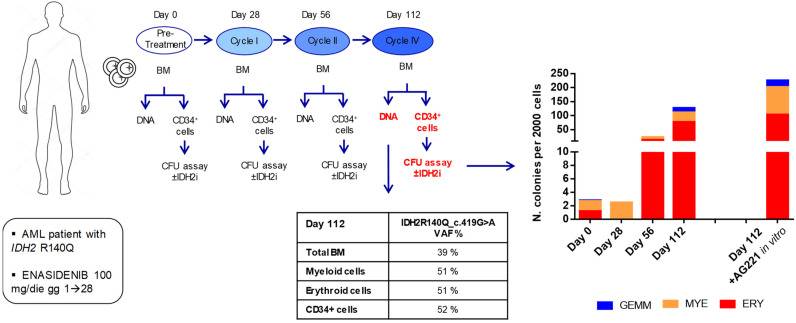
Enasidenib treatment induces the progressive improvement of CFU ability in primary CD34+ cells carrying the *IDH2*-R140Q mutation. Experimental scheme (left) and stacked columns chart (right) of colonies that rose after 14 days from CD34+ cells isolated from the patient’s BM before the start of treatment (Day 0) and at I, II, and IV cycles of enasidenib therapy (Days 28, 56, and 112, respectively). The table in the middle shows *IDH2*-R140Q VAF obtained by targeted NGS on DNA extracted from either bulk BM or isolated cell subpopulations at cycle IV/Day 112 of treatment.

In order to evaluate the dynamics of fitness of HSPCs in terms of their ability to form colonies, we isolated and studied CD34+ cells from the bone marrow of a patient with *IDH2*-R140Q-mutated AML, and we had the opportunity to follow under treatment with enasidenib. Specifically, we performed CFU assays with AML patient-derived CD34+ cells either at baseline or at the end of cycles I, II, and IV. Moreover, with cells obtained after the IV cycle, we explored the effect of adding pharmacological AG-221 inhibition in vitro during culture to assess whether maintaining the drug exposure in culture could enhance their CFU ability. Colonies were evaluated at 14 days as described above (Figure 4).

Strikingly, we observed that in vivo treatment with enasidenib induced a progressive increase, over time, in the CFU capacity of the patient-derived CD34+ cells, whose fitness appeared progressively improved in terms of developing mature hematopoietic precursors across the different lineages, a phenomenon that was further strengthened when drug exposure was maintained in vitro in semi-solid culture (Figure 4). This occurred in parallel with the hematologically complete remission of the patient.

Remarkably, the NGS analysis performed after cycle IV, with respect to the diagnosis, showed the persistence of the *IDH2*-R140Q mutation, on the one hand, in the bulk, as well as in isolated myeloid and erythroid cells (variant allele frequency, VAF = 51% for both subpopulations), and on the other hand, in the CD34+ subpopulation (VAF = 52%) used for the CFU assay (Figure 4).

The VAF values found in the different cell subpopulations suggest that the entire hemopoiesis, including the pool of CD34+ cells, were clonal (*IDH2*-R140Q positive), and that the improvement in the fitness of HSPC CD34+ relies on the protracted inhibitory action of enasidenib, which unleashes the block of differentiation mediated by the *IDH2*-R140Q mutation.

Despite being observed in a single patient, these findings are in line with clinical observations that complete remission in patients treated with the IDH2 inhibitor is achieved after more cycles of therapy (mean n = 5 cycles) without undergoing bone marrow aplasia; these results are also associated with the cell differentiation of bone marrow cells in the absence of clearance of the *IDH2* mutation [26,41].

## 4. Discussion

Here, we report our experimental findings on how *IDH1/2* mutations and their pharmacological inhibitions affect human hematopoietic stem cell fitness. Our model is the first to represent AML-associated *IDH1/2* mutation modeling in the human system. Indeed, from the review of the models available so far, it emerges that the early effects of *IDH1/2* mutations, particularly in human hematopoiesis, are mostly unexplored (Supplementary Table S2). 

This work, as well as our previous preliminary reports [42,43], show for the first time that expressing the *IDH1*-R132H or *IDH2*-R140Q mutant is sufficient to drive a complete block of hematopoietic cell differentiation in primary human HSPCs in vitro; this block can be efficiently released by the specific inhibitor, restoring differentiation ability across all lineages. Despite the relatively low number of normal donors (n = 7, for *IDH1*-R132H; n = 6, for *IDH2*-R140Q) analyzed in our study, the results are very consistent among the different samples, supporting the need for further studies to more deeply explore our preliminary findings.

The block of differentiation represents a known early event in leukemogenesis, which is generally associated with increased self-renewal activity, forming the backbone for the development of leukemia upon the acquisition of other genetic alterations and blast proliferation [2].

In vitro cellular models of *IDH* mutations, either human or murine, highlight a significant increase in DNA methylation in *IDH1/2* mutant-expressing cells [24], in keeping with the hypermethylation signature observed in AML patients [24], and is supported by experimental data showing impaired myeloid differentiation [24,35,36] (Supplementary Materials).

DNA hypermethylation in hematopoietic progenitors has also been reported via in vivo studies in conditional knock-in mice with the *IDH1*-R132H mutation, associated with a significant increase in the pool of hematopoietic precursors and self-renewal capacity [30]. Data emerging from our CD34+ HSPC model of the *IDH1*-R132H mutation are in contrast with this previous study of the murine model. Indeed, in human CD34+ HSPCs from healthy donors, the expression of the mutated *IDH1* transgene induces a significant block of differentiation that was not observed in the Sasaki et al. mouse model [30].

The expansion of early hematopoietic progenitors and increased self-renewal were also detected in a model of transgenic mice expressing *IDH2*-R140Q in the hematopoietic system [31], where, in keeping with our results, a potent block of differentiation was also observed. However, the block primarily affected erythroid colonies, which were severely reduced, while myeloid colonies were not compromised. Instead, the marked block of differentiation that we show—following the expression of the *IDH2* mutation in human CD34+ HSPCs—features trilinear myeloid involvement, consistent across the different donors (Figure 3), suggesting that this is a peculiar effect in the human system.

Indeed, strikingly, our findings are in line with the recent report from Landberg et al. [44,45], obtained in human CD34+ HSPCs but edited by the CRISPR/Cas9 and AAV6-mediated homology-directed repair to express *IDH1*-R132H or *IDH2*-R140Q mutations. A pronounced reduction in the colony formation and differentiation capacity triggered by both *IDH* mutations as well as the ability of ivosidenib to restore the block of differentiation driven by *IDH1*-R132H were shown. The report from Landberg et al. [44] and our findings highlight the direct involvement of the AML-associated *IDH1*-R132H and *IDH2*-R140Q mutations in the hematopoietic differentiation process.

In this sense, the revertant actions of the specific inhibitors described in our (and other studies) underline the dependence of the cellular differentiation of hematopoietic precursors on the specific genetic mutation. The drug-induced ‘unblock’ of differentiation is unveiled in our experimental conditions in vitro using the CFU assay, showing that the ability of hematopoietic precursors harboring the *IDH1/2* mutation consistently recover their potential to give rise to heterogeneous colonies similar to the negative control (Figure 2 and Figure 3). These findings may suggest that in patients affected by *IDH*-mutated AML, treatment with the specific inhibitor leads to a phase of differentiation of leukemic cells and clonal trilineage hemopoietic recovery with peripheral blood count improvement, which occurs in clinically responding patients, who also show progressive transfusion independence, as reported in seminal clinical studies [26,27,41].

Our study on the fitness dynamics of primary HSPCs from an *IDH2*-mutated AML patient undergoing enasidenib treatment, despite being limited to a single case, appears to be in line with these findings and clinical reports.

## 5. Conclusions

Our report highlights how *IDH1/2* mutations frequently found in acute myeloid leukemia act at the level of hematopoietic precursors in the human system by inducing a block of differentiation. The block involves all the hematopoietic myeloid lineages and is specifically reversed by the targeted therapy. Furthermore, we provide evidence that the specific inhibitor acts progressively on the colony-forming ability of HSPCs in patients in vivo, and sustains trilinear hematopoiesis over time in vivo, leading to hematological recovery and complete hematological remission. Our model also serves as a suitable model for further mechanistic studies to explore the role of *IDH1/2* mutations in human leukemogenesis.

## Data Availability

The original contributions presented in the study are included in the article/Supplementary Material, further inquiries can be directed to the corresponding author.

## Supplementary Materials

The following supporting information can be downloaded at: https://www.mdpi.com/article/10.3390/cancers16152675/s1, Figure S1: Flow cytometry-based sorting strategy for human CD34+ transduced with *IDH* transgenes; Table S1: Experimental conditions of HSPC CD34+ cells from 13 healthy donors; Supplemental information. Description of the models available in the literature; Table S2: Hematopoiesis models of IDH1/2 AML-associated mutations.

## References

[1] Ley T. (2013). Genomic and epigenomic landscapes of adult de novo acute myeloid leukemia. N. Engl. J. Med..

[2] Gilliland D. (2002). Molecular genetics of human leukemias: New insights into therapy. Semin. Hematol..

[3] McMurry H., Fletcher L., Traer E. (2021). Idh inhibitors in aml-promise and pitfalls. Curr. Hematol. Malig. Rep..

[4] Parsons D.W., Jones S., Zhang X., Lin J.C., Leary R.J., Angenendt P., Mankoo P., Carter H., Siu I.M., Gallia G.L. (2008). An integrated genomic analysis of human glioblastoma multiforme. Science.

[5] Yan H., Parsons D.W., Jin G., McLendon R., Rasheed B.A., Yuan W., Kos I., Batinic-Haberle I., Jones S., Riggins G.J. (2009). Idh1 and idh2 mutations in gliomas. N. Engl. J. Med..

[6] Mardis E.R., Ding L., Dooling D.J., Larson D.E., McLellan M.D., Chen K., Koboldt D.C., Fulton R.S., Delehaunty K.D., McGrath S.D. (2009). Recurring mutations found by sequencing an acute myeloid leukemia genome. N. Engl. J. Med..

[7] Green A., Beer P. (2010). Somatic mutations of idh1 and idh2 in the leukemic transformation of myeloproliferative neoplasms. N. Engl. J. Med..

[8] Thol F., Weissinger E.M., Krauter J., Wagner K., Damm F., Wichmann M., Göhring G., Schumann C., Bug G., Ottmann O. (2010). Idh1 mutations in patients with myelodysplastic syndromes are associated with an unfavorable prognosis. Haematologica.

[9] Amary M.F., Bacsi K., Maggiani F., Damato S., Halai D., Berisha F., Pollock R., O'Donnell P., Grigoriadis A., Diss T. (2011). Idh1 and idh2 mutations are frequent events in central chondrosarcoma and central and periosteal chondromas but not in other mesenchymal tumours. J. Pathol..

[10] Murugan A.K., Bojdani E., Xing M. (2010). Identification and functional characterization of isocitrate dehydrogenase 1 (idh1) mutations in thyroid cancer. Biochem. Biophys. Res. Commun..

[11] Papaemmanuil E., Gerstung M., Bullinger L., Gaidzik V.I., Paschka P., Roberts N.D., Potter N.E., Heuser M., Thol F., Bolli N. (2016). Genomic classification and prognosis in acute myeloid leukemia. N. Engl. J. Med..

[12] Döhner H., Wei A.H., Appelbaum F.R., Craddock C., DiNardo C.D., Dombret H., Ebert B.L., Fenaux P., Godley L.A., Hasserjian R.P. (2022). Diagnosis and management of aml in adults: 2022 recommendations from an international expert panel on behalf of the eln. Blood.

[13] Metzeler K.H., Herold T., Rothenberg-Thurley M., Amler S., Sauerland M.C., Görlich D., Schneider S., Konstandin N.P., Dufour A., Bräundl K. (2016). Spectrum and prognostic relevance of driver gene mutations in acute myeloid leukemia. Blood.

[14] Silva P., Neumann M., Schroeder M.P., Vosberg S., Schlee C., Isaakidis K., Ortiz-Tanchez J., Fransecky L.R., Hartung T., Türkmen S. (2017). Acute myeloid leukemia in the elderly is characterized by a distinct genetic and epigenetic landscape. Leukemia.

[15] Ogawara Y., Katsumoto T., Aikawa Y., Shima Y., Kagiyama Y., Soga T., Matsunaga H., Seki T., Araki K., Kitabayashi I. (2015). Idh2 and npm1 mutations cooperate to activate hoxa9/meis1 and hypoxia pathways in acute myeloid leukemia. Cancer Res..

[16] Molenaar R.J., Maciejewski J.P., Wilmink J.W., van Noorden C.J.F. (2018). Wild-type and mutated idh1/2 enzymes and therapy responses. Oncogene.

[17] Marcucci G., Maharry K., Wu Y.Z., Radmacher M.D., Mrózek K., Margeson D., Holland K.B., Whitman S.P., Becker H., Schwind S. (2010). Idh1 and idh2 gene mutations identify novel molecular subsets within de novo cytogenetically normal acute myeloid leukemia: A cancer and leukemia group b study. J. Clin. Oncol..

[18] Im A.P., Sehgal A.R., Carroll M.P., Smith B.D., Tefferi A., Johnson D.E., Boyiadzis M. (2014). Dnmt3a and idh mutations in acute myeloid leukemia and other myeloid malignancies: Associations with prognosis and potential treatment strategies. Leukemia.

[19] Martelli M.P., Martino G., Cardinali V., Falini B., Martinelli G., Cerchione C. (2020). Enasidenib and ivosidenib in aml. Minerva Med..

[20] Ward P.S., Patel J., Wise D.R., Abdel-Wahab O., Bennett B.D., Coller H.A., Cross J.R., Fantin V.R., Hedvat C.V., Perl A.E. (2010). The common feature of leukemia-associated idh1 and idh2 mutations is a neomorphic enzyme activity converting alpha-ketoglutarate to 2-hydroxyglutarate. Cancer Cell.

[21] Dang L., Su S.M. (2017). Isocitrate dehydrogenase mutation and (r)-2-hydroxyglutarate: From basic discovery to therapeutics development. Annu. Rev. Biochem..

[22] Dang L., White D.W., Gross S., Bennett B.D., Bittinger M.A., Driggers E.M., Fantin V.R., Jang H.G., Jin S., Keenan M.C. (2009). Cancer-associated idh1 mutations produce 2-hydroxyglutarate. Nature.

[23] Lu C., Ward P.S., Kapoor G.S., Rohle D., Turcan S., Abdel-Wahab O., Edwards C.R., Khanin R., Figueroa M.E., Melnick A. (2012). Idh mutation impairs histone demethylation and results in a block to cell differentiation. Nature.

[24] Figueroa M.E., Abdel-Wahab O., Lu C., Ward P.S., Patel J., Shih A., Li Y., Bhagwat N., Vasanthakumar A., Fernandez H.F. (2010). Leukemic idh1 and idh2 mutations result in a hypermethylation phenotype, disrupt tet2 function, and impair hematopoietic differentiation. Cancer Cell.

[25] Falini B., Spinelli O., Meggendorfer M., Martelli M.P., Bigerna B., Ascani S., Stein H., Rambaldi A., Haferlach T. (2019). Idh1-r132 changes vary according to npm1 and other mutations status in aml. Leukemia.

[26] Stein E.M., DiNardo C.D., Pollyea D.A., Fathi A.T., Roboz G.J., Altman J.K., Stone R.M., DeAngelo D.J., Levine R.L., Flinn I.W. (2017). Enasidenib in mutant idh2 relapsed or refractory acute myeloid leukemia. Blood.

[27] DiNardo C.D., Stein E.M., de Botton S., Roboz G.J., Altman J.K., Mims A.S., Swords R., Collins R.H., Mannis G.N., Pollyea D.A. (2018). Durable remissions with ivosidenib in idh1-mutated relapsed or refractory aml. N. Engl. J. Med..

[28] Golub D., Iyengar N., Dogra S., Wong T., Bready D., Tang K., Modrek A.S., Placantonakis D.G. (2019). Mutant isocitrate dehydrogenase inhibitors as targeted cancer therapeutics. Front. Oncol..

[29] Miles L.A., Bowman R.L., Merlinsky T.R., Csete I.S., Ooi A.T., Durruthy-Durruthy R., Bowman M., Famulare C., Patel M.A., Mendez P. (2020). Single-cell mutation analysis of clonal evolution in myeloid malignancies. Nature.

[30] Sasaki M., Knobbe C.B., Munger J.C., Lind E.F., Brenner D., Brüstle A., Harris I.S., Holmes R., Wakeham A., Haight J. (2012). Idh1(r132h) mutation increases murine haematopoietic progenitors and alters epigenetics. Nature.

[31] Kats L.M., Reschke M., Taulli R., Pozdnyakova O., Burgess K., Bhargava P., Straley K., Karnik R., Meissner A., Small D. (2014). Proto-oncogenic role of mutant idh2 in leukemia initiation and maintenance. Cell Stem Cell.

[32] Chen C., Liu Y., Lu C., Cross J.R., Morris J.P.T., Shroff A.S., Ward P.S., Bradner J.E., Thompson C., Lowe S.W. (2013). Cancer-associated idh2 mutants drive an acute myeloid leukemia that is susceptible to brd4 inhibition. Genes Dev..

[33] Marshall A., Kasturiarachchi J., Datta P., Guo Y., Deltcheva E., James C., Brown J., May G., Anandagoda N., Jackson I. (2020). Mir142 loss unlocks idh2(r140)-dependent leukemogenesis through antagonistic regulation of hox genes. Sci. Rep..

[34] Gruber E., So J., Lewis A.C., Franich R., Cole R., Martelotto L.G., Rogers A.J., Vidacs E., Fraser P., Stanley K. (2022). Inhibition of mutant idh1 promotes cycling of acute myeloid leukemia stem cells. Cell Rep..

[35] Losman J.A., Looper R.E., Koivunen P., Lee S., Schneider R.K., McMahon C., Cowley G.S., Root D.E., Ebert B.L., Kaelin W.G. (2013). (r)-2-hydroxyglutarate is sufficient to promote leukemogenesis and its effects are reversible. Science.

[36] Wang F., Travins J., DeLaBarre B., Penard-Lacronique V., Schalm S., Hansen E., Straley K., Kernytsky A., Liu W., Gliser C. (2013). Targeted inhibition of mutant idh2 in leukemia cells induces cellular differentiation. Science.

[37] Shih A.H., Meydan C., Shank K., Garrett-Bakelman F.E., Ward P.S., Intlekofer A.M., Nazir A., Stein E.M., Knapp K., Glass J. (2017). Combination targeted therapy to disrupt aberrant oncogenic signaling and reverse epigenetic dysfunction in idh2- and tet2-mutant acute myeloid leukemia. Cancer Discov..

[38] Kats L.M., Vervoort S.J., Cole R., Rogers A.J., Gregory G.P., Vidacs E., Li J., Nagaraja R., Yen K.E., Johnstone R.W. (2017). A pharmacogenomic approach validates ag-221 as an effective and on-target therapy in idh2 mutant aml. Leukemia.

[39] Martelli M.F., Di Ianni M., Ruggeri L., Pierini A., Falzetti F., Carotti A., Terenzi A., Reisner Y., Aversa F., Falini B. (2014). Designed" Grafts for hla-haploidentical stem cell transplantation. Blood.

[40] Girard-Gagnepain A., Amirache F., Costa C., Lévy C., Frecha C., Fusil F., Nègre D., Lavillette D., Cosset F.L., Verhoeyen E. (2014). Baboon envelope pseudotyped lvs outperform vsv-g-lvs for gene transfer into early-cytokine-stimulated and resting hscs. Blood.

[41] Stein E.M., DiNardo C.D., Fathi A.T., Pollyea D.A., Stone R.M., Altman J.K., Roboz G.J., Patel M.R., Collins R., Flinn I.W. (2019). Molecular remission and response patterns in patients with mutant-idh2 acute myeloid leukemia treated with enasidenib. Blood.

[42] Ciaurro V., Pierangeli S., Falini B., Martelli M.P. Idh1-r132h expression drives in human normal cd34+ hematopoietic cells a block of differentiation released by the specific inhibitor ivosidenib. Proceedings of the XVI Congress of the Italian Society of Experimental Hematology.

[43] Pierangeli S., Ciaurro V., Donnini S., Milano F., Sabino M., Gionfriddo I., Ranieri R., Silvestri S., Tini V., Spinozzi G. Isocitrate dehydrogenases aml-associated point mutations drive a block of differentiation in human normal cd34+hematopoietic cells that is released by specific inhibitors. Proceedings of the XVII Congress of the Italian Society of Experimental Hematology.

[44] Landberg N., Koehnke T., Nakauchi Y., Fan A., Karigane D., Thomas D., Majeti R. (2022). Targeting idh1-mutated pre-leukemic hematopoietic stem cells in myeloid disease, including CCUS and AML. Blood.

[45] Landberg N., Köhnke T., Feng Y., Nakauchi Y., Fan A.C., Linde M.H., Karigane D., Lim K., Sinha R., Malcovati L. (2024). Idh1-mutant preleukemic hematopoietic stem cells can be eliminated by inhibition of oxidative phosphorylation. Blood Cancer Discov..

