# Supplementary material for: The Leukemic Isocitrate Dehydrogenase (IDH) 1/2 Mutations Impair Myeloid and Erythroid Cell Differentiation of Primary Human Hematopoietic Stem and Progenitor Cells (HSPCs)
Source: Cancers (Basel). 2024 Jul 27;16(15):2675. doi: 10.3390/cancers16152675 (PMC11312189; doi:10.3390/cancers16152675)
Supplement: Supplementary file 1 [file cancers-16-02675-s001.zip › cancers-3095638-supplementary.pdf]

Article

The leukemic *isocitrate dehydrogenases (IDH)* 1/2 mutations impair myeloid and erythroid cell differentiation of primary human hematopoietic stem and progenitor cells (HSPC).

Sara Pierangeli[1&], Serena Donnini[1&], Valerio Ciaurro[2], Francesca Milano[1], Valeria Cardinali[1,3], Sofia Sciabolacci[3], Gaetano Cimino[1,3], Ilaria Gionfriddo[1], Roberta Ranieri[1], Sabrina Cipriani[1], Eleonora Padiglioni[1], Roberta Iacucci Ostini[3], Tiziana Zei[3], Antonio Pierini[1,3] and Maria Paola Martelli[1,3*]

1 Hematology and Clinical Immunology Section, Department of Medicine and Surgery, Center for Hemato-Oncological Research (CREO), University of Perugia, Perugia, Italy
2 MD Anderson Cancer Center, University of Texas, Texas, USA
3 Hematology Department, 'Santa Maria della Misericordia' Perugia Hospital, Perugia, Italy

* Correspondence: maria.martelli@unipg.it

& These authors contributed equally to this work

Table S1. Experimental conditions of HSPC CD34+ cells from 13 healthy donors

	DONORS	CONDITIONS				
		EMPTY	WT	WT+AG-120	R132H	R132H+AG-120
IDH1	Donor 1	x	x	x	x	x
	Donor 2	x	x	x	x	x
	Donor 3	x	x	x	x	x
	Donor 4	x	x		x	
	Donor 5	x	x		x	
	Donor 6	x	x		x	
	Donor 7	x			x	

	DONORS	CONDITIONS				
		EMPTY	WT	WT+AG-221	R140Q	R140Q+AG-221
IDH2	Donor 8	x	x	x	x	x
	Donor 9	x	x	x	x	x
	Donor 10	x	x	x	x	x
	Donor 11	x	x	x	x	x
	Donor 12	x	x		x	
	Donor 13	x	x		x	

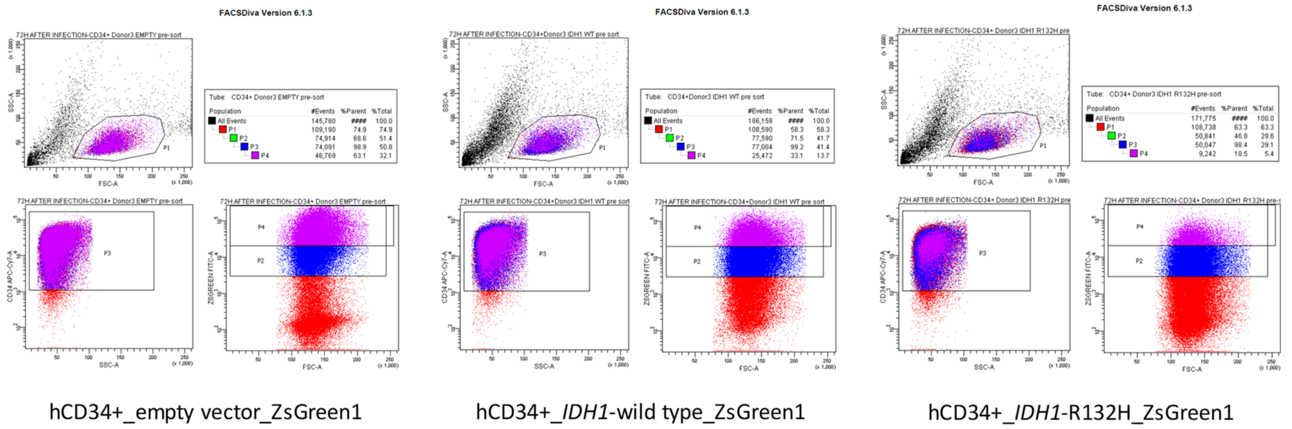

Figure S1. Flow cytometry-based **s**orting strategy for human CD34+ transduced with *IDH* transgene. Example of dot plot related to *IDH1* experimental set generated by cell sorter software. Each plot illustrates FSC/SSC physical parameters (up), CD34 positivity (down/left) and ZsGreen1 positivity (down/right) concerning empty vector (left panel), wild-type gene (middle panel) and mutated gene (right panel). Transduced bright ZsGreen1+/CD34+ double positive cells gated in P4 were sorted for CFU assay.

Supplemental information.

1. Description of the models available in the literature

1.1. In Vitro Models of IDH mutations

Figueroa et al. [24]developed several *in vitro* cell models to explore the role of *IDH* mutations in leukemogenesis: transiently transfected 293T human cell line expressing *IDH1*-R132H or *IDH2*-R172K mutant, mouse myeloid progenitor 32D cells stably expressing *IDH2*-R172K mutant and murine primary bone marrow cells bearing *IDH1*-R132H or *IDH2*-R172K mutations. All of these models showed a significant increase in DNA methylation compared to control cells expressing *IDH* wild-type genes; this result was in keeping with the observed association of *IDH1/2* mutations with hypermethylation signature in a large cohort of *de novo* AML patients. Stable expression of mutant *IDH2* resulted in an important increase in c-Kit stemness marker expression both in 32D cells and in the murine primary bone marrow cells; in the latter, a reduced expression of the mature myeloid markers (Gr-1 and Mac-1) was also observed. Ultimately, the *Figueroa et al.*[24] study establishes that *IDH1/2* mutant expression may specifically alter DNA methylation in AML cells and this can correlate with their role in impairing myeloid differentiation as observed in the established *in vitro* models.

Losman et al. [35]chose the TF-1 human erythroleukemia cell line as a model; these cells are GM-CSF-dependent and retain the ability to differentiate in response to erythropoietin (EPO). When stably infected by lentiviral vectors encoding *IDH1*-R132H, TF-1 cells became cytokine-independent and no longer differentiated upon EPO at the first steps after infection. In this study the inhibitory effect of mutant *IDH* on hematopoietic differentiation was confirmed on the SCF ER-Hoxb8 cell model, immortalized granulocyte-macrophage progenitor cells derived from primary murine bone marrow cells. In the presence of estrogen, these cells express a functional ER-Hoxb8 fusion protein that promotes their survival and proliferation. Upon estrogen withdrawal, the

cells differentiate and up-regulate expression of the monocytic markers CD11b/Mac1 and Gr1. Stable expression of *IDH1*-R132H transgene in SCF ER-Hoxb8 cells, however, blunted their differentiation in response to estrogen withdrawal. Therefore, growth factor/hormone independence and impaired differentiation seem to be the two fundamental steps in leukemic transformation promoted by IDH mutant in *Losman et al.* models.

Wang et al. [36] developed a small molecule IDH2-R140Q selective inhibitor (AGI-6780) and used human TF-1 erythroleukemia cell line expressing IDH2-R140Q to study its biological effect. Expression of the mutant protein induced a large production of 2HG, a morphological change in TF-1 cells concomitantly to increase in vimentin expression, and decreased CD38; this evidence suggested that IDH2 mutant expression could induce a more immature phenotype, shifting TF-1 cells to an earlier stage, blocking hematopoietic differentiation. Furthermore, EPO treatment of TF-1 cells bearing *IDH2*-R140Q failed to induce the differentiation and the expression of genes upregulated during erythropoiesis (i.e. *HBG* and *KLF1*); importantly, pharmacological inhibition by AGI-6780 could revert both these phenomena. These results were in keeping with studies of *Losman et al.* representing the counterpart of the *IDH1*-R132H mutation in the same *in vitro* model.

1.2. In Vivo Models of IDH mutations

Sasaki et al. [30]developed and characterized a conditional knock-in (KI) mice with *IDH1*-R132H mutation expressed in all hematopoietic cells (Vav-KI mice) or specifically in cells of the myeloid lineage (LysM-KI mice). The LysM-KI mouse model showed increased numbers of early hematopoietic progenitors and developed splenomegaly, signals of dysfunctional bone marrow niche and, main, hypermethylated histones and changes in DNA methylation similar to those observed in human *IDH1/2* mutant AML[24]. The evaluation of HPSC compartments in older LysM-KI mice showed a bone marrow with fewer mature cells and more immature elements. In CFU assays, mice showed statistically normal production of CFU-GEMM, CFU-GM, CFU-G and CFU-M colonies. Serial plating experiments showed that, whereas control BM cells stopped proliferating, LysM-KI BM cells continued to grow at an exponential rate. The DNA-methylation analysis of sorted LSK (Lineage–Sca-1+cKit+) cells from young LysM-KI showed a significantly greater proportion of highly methylated CpG sites compared to control mice. DNA-methylation involved several pathways concerning hematopoietic cell proliferation and differentiation, leukemogenesis and leukemic stem cell maintenance. *Sasaki et al.* [30]therefore did not observe a block of differentiation and of CFU ability in mouse model with the *IDH1*-R132H mutation; neverthless they detected a significant increase in the pool of hematopoietic precursors and self-renewal capacity associated to the typical hypermethylation profile.

Kats et al. [31] generated transgenic mice expressing *IDH2*-R140Q in an on/off- and tissue-specific manner using a tetracycline-inducible system, in order to establish the impact of *IDH2* mutation on normal HSPC as well as lineage development *in vivo*. Short-term analyses of hematopoiesis in transgenic *IDH2*-R140Q animals showed no differences in peripheral blood cell counts and, likewise, there were no alterations in the numbers of hematopoietic stem cells, myeloid progenitors, mature B, T, or myeloid cells in the bone marrow. The effect of this mutation evaluated at longer-term showed an increase in the number of LSK cells in the BM. Evaluating differentiation of primary hematopoietic cells by methylcellulose differentiation assay on KSL cells from *IDH2*-R140Q mice a potent block of differentiation was observed. Especially erythroid colonies were severely reduced, while the number of myeloid colonies was unaffected. Moreover, in serial plating experiments *IDH2*-R140Q cells

formed more colonies in the second plating when compared with controls. The block of differentiation induced by *IDH2* mutation in Kats's model could be reversed and differentiation of mutant cells was restored either upon *IDH2*-R140Q doxycycline de-induction or upon AGI-6780 treatment. In conclusion, the expression of the *IDH2* mutation in this mouse model is sufficient to induce defects in differentiation of primary hematopoietic cells with restriction to the erythroid lineage and is reversible by the specific pharmacological inhibition. Like *IDH1*, also *IDH2* mutation is associated with an expansion of hematopoietic stem cells with a consequent increase in their self-renewal capacity.

1.3. In Vivo Models of IDH mutations combined with other genetic lesions

Chen et al. [32] and *Shih et al.* [37] demonstrated that *IDH2*-R140Q and *IDH2*-R172K mutants can be potent oncogenes in mice, acting as driver mutations that cooperate with the *FLT3*-ITD or the *NRAS*-G12D mutation to promote aggressive AML. They showed that *IDH2* mutants are required for sustained 2-HG production and leukemia maintenance; moreover, suppression of IDH2 mutant levels and its neo-morphic activity confirmed to trigger myeloid differentiation.

Marshall et al. [33] with their work demonstrated that *IDH* mutation can cooperate with other "not classic" alterations, creating a mouse model with *Mir142* loss-of- function in which *IDH2*-R140Q mutation synergizes to promote leukemogenesis.

Gruber et al. [34], on their side, developed a multigenic AML mouse model with inducible *IDH1*-R132H mutation in its prevalent combination with *DNMT3A*-R882H and *NRAS*-G12D. Inhibition of mutant *IDH1* in this model promoted AML differentiation and prolonged survival of mice. Similarly, *Kats et al.* [38] created a combinatorial mouse model with *IDH2*-R140Q, *DNMT3A*-R882H and *NRAS*-G12D that develops AML. Silencing of *IDH2*-R140Q expression again led to terminal myeloid differentiation.

Table S1. Hematopoiesis models of *IDH1/2* AML-associated mutations.

REFERENCE#	IDH mutations	MODELS	OBSERVATIONS
		in vitro*	
Figueroa et al. [24]	*IDH1*-R132H or *IDH2*-R172K/R140Q	293T human embryo kidney cell line transiently expressing *IDH1/2* mutations<br>32D mouse myeloid progenitor cell line stably expressing *IDH2* mutations<br>Murine primary bone marrow cells with *IDH1/2* mutations	*IDH1/2* mutants alter DNA methylation and impair myeloid differentiation
Losman et al. [35]	*IDH1*-R132H	TF-1 human erythroleukemia cell line stably expressing *IDH1*-R132H<br>SCF ER-Hoxb8 murine granulocyte/macrophage progenitor cells stably expressing *IDH1*-R132H	*IDH1* mutant promote leukemogenesis by growth factor/hormone independence and impaired differentiation
Wang et al. [36]	*IDH2*-R140Q	TF-1 human erythroleukemia cell line expressing *IDH2*-R140Q	*IDH2* mutant induce an immature phenotype blocking differentiation
		in vivo	
Sasaki et al. [30]	*IDH1*-R132H	Knock-in (KI) mice expressing *IDH1*-R132H mutation in all hematopoietic cells (Vav-KI mice) or in cells of myeloid lineage (LysM-KI mice)	*IDH1* mutant doesn't block differentiation and increases hematopoietic precursors
Kats et al. [31]	*IDH2*-R140Q	Transgenic mice expressing *IDH2*-R140Q in an on/off- and tissue-specific manner by a tetracycline- inducible system	*IDH2* mutant blocks only erythroid lineage differentiation and increases self-renewal capacity

Chen et al. [32]<br>*Shih et al. [37]*	*IDH2*-R140Q /R172K	Mosaic mouse model *FLT3*-ITD or *NRAS*-G12D mutated and transduced with *IDH2* mutant	*IDH2* mutants sustain leukemia maintenance and their suppression restores differentiation
Marshall et al. [33]	*IDH2*-R140Q /R172K	Mouse model with Mir142 loss-of- function and IDH2-R140Q mutation	*IDH2* mutation cooperates with peculiar genetic alteration for leukemogenesis
Gruber et al. [34]	*IDH1*-R132H	Multigenic mouse model with inducible *IDH1*-R132 mutation in combination with *DNMT3A*-R882H and *NRAS*-G12D	*IDH1* mutant, together with other alterations, if inhibited promote AML differentiation
Kats et al. [38]	*IDH2*-R140Q	Combinatorial mouse model with *IDH2*-R140Q, *DNMT3A*-R882H and *NRAS*-G12D mutations	*IDH2* mutant silencing leads to terminal myeloid differentiation

*cell line source is reported in the original cited reference

see reference citation in the main text
